# Application of Managed Entry Agreements for Innovative Therapies in Different Settings and Combinations: A Feasibility Analysis

**DOI:** 10.3390/ijerph17228309

**Published:** 2020-11-10

**Authors:** Rick A Vreman, Thomas F Broekhoff, Hubert GM Leufkens, Aukje K Mantel-Teeuwisse, Wim G Goettsch

**Affiliations:** 1 Division of Pharmacoepidemiology & Clinical Pharmacology, Utrecht Institute for Pharmaceutical Sciences (UIPS), Utrecht University, 3584 CG Utrecht, The Netherlands; r.a.vreman@uu.nl (R.A.V.); t.f.broekhoff@students.uu.nl (T.F.B.); H.G.M.Leufkens@uu.nl (H.G.M.L.); A.K.Mantel@uu.nl (A.K.M.-T.); 2 National Health Care Institute (ZIN), 1112 ZA Diemen, The Netherlands

**Keywords:** managed entry agreement, reimbursement, payment, pricing, value-based pricing, health technology assessment, coverage with evidence development, pay-for-performance, outcome-based payment

## Abstract

The reimbursement of expensive, innovative therapies poses a challenge to healthcare systems. This study investigated the feasibility of managed entry agreements (MEAs) for innovative therapies in different settings and combinations. First, a systematic literature review included studies describing used or conceptual agreements between payers and manufacturers (i.e., MEAs). Identical and similar MEAs were clustered and data were extracted on their benefits and limitations. A feasibility assessment was performed for each individual MEA based on how it could be applied (financial/outcome-based), on what level (individual patients/target population), in which payment setting (centralized pricing and reimbursement authority yes/no), for what type of therapies (one-time/chronic), within what payment structures, and whether combinations with other MEAs were feasible. The literature search ultimately included 82 papers describing 117 MEAs. After clustering, 15 unique MEAs remained, each describing one or multiple similar agreements. Four of those entailed payment structures, while eleven entailed agreements between payers and manufacturers regarding price, usage, and/or evidence generation. The feasibility assessment indicated that most agreements could be applied throughout the different settings that were assessed and could be applied in different payment structures and in combination with multiple other agreements. The potential to combine multiple agreements leads to a multitude of different reimbursement mechanisms that may manage the price, usage, payment structure, and additional conditions for an innovative therapy. This overview of the feasibility of combinations of MEAs can help decision-makers construct a reimbursement mechanism most suited to their preferences, the type of therapy under evaluation, and the applicable healthcare system.

## 1. Introduction

To manage the costs associated with expensive, innovative therapies, a variety of measures can be taken by the entity or entities responsible for pricing and reimbursement (hereafter referred to as payers) [1,2]. These measures aim to control the financial risks associated with the introduction of the therapy by controlling the price of a therapy, its budget impact, its payment structure, or its usage. Any agreement beyond a yes/no decision on reimbursement between the marketing authorization holder (hereafter called manufacturer) of a therapy and a payer can be called a managed entry agreement (MEA) [1].

A recent report about performance-based MEAs from the Organization for Economic Co-operation and Development (OECD) outlined that outcome-based agreements have been tried in multiple settings but often without a systematic approach to their selection and application, and with mixed results [1]. MEAs are usually broken down into purely financial agreements (e.g., simple discounts) and outcome-based agreements (e.g., pay-for-performance) [1,2,3,4]. Experience with financial agreements is extensive, while experience with outcome-based agreements is more limited [1,5]. The interpretation of the results from outcome-based agreements is further complicated by the fact that there are many different ways to implement them. For example, a pay-for-performance agreement entails payment for a specific result. However, this result can be measured on an individual level or on a population level, and the payment can be structured so that the therapy is paid upfront with rebates when a result is not achieved or the therapy can be paid only after the result has been achieved [1,2,3,4]. Though MEAs are thus inherently linked to payment structures, literature on MEAs varies in whether they include payment structures, and the distinction between payment structures and other agreements is often unclear [1,2]. Some of the key findings of the OECD report are that it is essential to define a strategy to guide the use of MEAs, and that it is key to implement a governance framework that ensures the transparency of processes.

The consequences of the lack of structured guidance on MEAs is exemplified by the introduction of potentially curative cell and gene therapies [6]. These therapies may be curative after a single treatment and come with high costs. The immediate budget impact is high, and while their benefits may last a lifetime, often long-term clinical data are not available at their introduction [2,7]. These high uncertainties have led payers to limit the use of these potentially (cost-)effective technologies. Therefore, payers need methods to appropriately manage the price and payment structures and balance these with the uncertainties associated with the available evidence.

In the U.S.—in a healthcare system with a combination of governmental and private payers—the Financing of Cures in the U.S. (FOCUS) initiative from the Massachusetts Institute of Technology (MIT) aims to provide structured guidance for these one-time curative therapies [8]. The FOCUS project used research, stakeholder assessments, and workshops to construct a toolbox that includes suggestions for the most promising strategies for introducing curative therapies in the U.S. These strategies all entail a combination of pricing agreements with a payment structure, sometimes combined with strategies for payers to mitigate risks outside their agreements with the manufacturer (e.g., by collaborating with other insurers to mitigate catastrophic costs).

Besides the impact that the type of therapy has on the feasibility of some MEAs, the way in which healthcare is organized and funded and the way decisions are made differ between countries and may impact the feasibility of some strategies [9]. Therefore, it is important to know which entry agreements exist, how they can be applied (financial or outcome-based), the level at which they can be applied (individual patient or target population level), what therapies they are tailored to (one-time or chronic), what payment system characteristics may be relevant (centralized pricing and reimbursement authority or not), and which agreements can or cannot easily be combined, either practically or conceptually.

This study aims to provide insight into the feasibility of MEAs in the different settings described above. First, a systematic review provides a comprehensive overview of potential MEAs. Second, a feasibility analysis based on the benefits and limitations of included MEAs provides insight into their applicability and the potential for combining them.

## 2. Materials and Methods

This study investigated the applicability of MEAs between payers and manufacturers for innovative therapies, for a set of MEAs obtained through a systematic review. The applicability of MEAs (applied as a financial or outcome-based agreement, on an individual patient or target population level) and their benefits and limitations were reviewed. Based on those, the feasibility of each agreement was linked to the type of therapy (chronic vs. one-time) and the type of payment system (centralized vs. decentral pricing and reimbursement authority), as well as to possible payment structures. The possibilities for combining agreements was also assessed.

### 2.1. Systematic Literature Review

For the systematic review, the Preferred Reporting Items for Systematic Reviews and Meta-Analyses (PRISMA) statement guidelines were followed [10]. A systematic literature search was conducted in PubMed and Embase on 15 February 2019. The full search strategy for both databases can be found in Appendix A. The search string contained two elements, each of which was broadly defined to be as inclusive as possible. The first element defined that searched papers should relate to reimbursement, economics, or costs. The second element defined that it had to relate to innovative or new therapies. Full papers (excluding abstracts) were included if they were published in a peer-reviewed journal, describing theoretical or conceptual design and/or practical implementation of an agreement between a payer and a manufacturer, written in English, and published from 2000 onward. The year 2000 was chosen to make sure included reimbursement mechanisms would still be applicable. No restrictions were applied regarding the type of therapy (e.g., drugs or devices) or the country setting (higher- vs. lower-income) The search strategy was first conceived in PubMed and later translated to Embase. The aim of the selection criteria was to achieve saturation regarding possible agreements.

After the search, duplicate records were removed using Mendeley Desktop 1.19.3 (Elsevier, New York, NY, USA). To come to a final selection of articles, first, the titles were screened on containing at least one keyword related to the specified search, listed in Appendix A. If a study did not contain any of the listed keywords, but the title still implied a focus on reimbursement of innovative therapies, it was also included. All other studies were excluded. Second, abstracts were read by two authors, and studies were excluded if they did not focus on MEAs between a payer and manufacturer. Third, full texts were screened for eligibility by two authors. Within the abstract and full-text screening, papers were only excluded when both authors excluded them. From the remaining included papers, references were screened, and when reference titles included one or more of the predefined keywords, the full text was screened for eligibility, ending up with the final cohort of included papers.

### 2.2. Data Extraction

From the final cohort of included papers, the following data were extracted: title; authors; journal; year of publication; the income level of the country they applied to country (World Bank classification high-income vs. other); whether they related to drugs, medical devices, or both; the suggested MEAs, their names, and descriptions; and the discussed benefits and limitations of those MEAs. Descriptions of MEAs were extracted from literature, but for those matching an MEA described in the Pharmaceutical Pricing and Reimbursement Information (PPRI) Glossary, the formal PPRI description was included [11].

For the MEAs suggested in the included papers, data were extracted on how they could be applied in four different types of settings, related to the following: type of agreement (purely financial vs. outcome-based), on what patient level (individual patient vs. target population), to which types of therapy (chronic vs. one-time), and in what type of payment system (central vs. decentral pricing and reimbursement authority). Central authority was defined as an entity that has the legal authority to enter into and enforce agreements. A sub-national authority, if not restricted by national laws or regulations, can represent such an entity. For example, individual states in the U.S. can enter into MEAs—Louisiana has made a subscription payment deal for hepatitis C treatment in June 2019.

### 2.3. Categorization and Clustering of Managed Entry Agreements

To define an exhaustive list of potential MEAs that each could be considered unique, each found MEA was categorized in a three-step process. The necessity of this approach is exemplified by a well-known managed entry tool; the reference pricing system. Reference pricing can include both internal (therapeutic) as well as external (international) reference pricing, but they are usually clustered into a single MEA called reference pricing [2,12,13]. To provide a set of unique MEAs—each including one or multiple similar agreements—we first took a set of categorized MEAs from a previous review [2]. Second, for each MEA included through our review, it was assessed whether its name or description matched or closely related to any of the predefined MEAs. Third, the remaining MEAs were assessed for similarity and subsequently clustered and given their own name. Similarity was assessed by two authors and was based on the name, definition, the goals, the methods employed, and the described benefits and limitations of the MEA. Discrepancies between authors were resolved by discussion until consensus was reached. When clustering MEAs, the data extracted for each of them (e.g., benefits and limitations) were aggregated. In regard to the example, the two types of reference pricing were ultimately clustered based on the great similarity between both their goals and applied methods.

For the purpose of this study, as described in the introduction and in line with previous studies [1,3,4], we considered payment structures (e.g., full upfront payment) separately from those MEAs that settle pricing of a therapy (e.g., value-based pricing), its application, or that capture evidence generation obligations or other conditions. Payment structures were included in the systematic literature review, data extraction, and clustering, but since payment structures are mutually exclusive, they were not included in the feasibility assessment as MEAs. Rather, it was assessed which payment structures could apply to each of the other MEAs.

### 2.4. Feasibility Analysis

After clustering the MEAs, their feasibility in different settings was assessed. Feasibility was defined as whether the included literature specified the applicability or a lack thereof of an MEA for each of the four predefined settings, to what payment structures the MEA could be connected, and the other MEAs with which the MEA could be combined. If literature did not clearly specify the feasibility of an MEA in a certain setting, it was interpreted from the information on benefits and limitations by the investigators. The resulting feasibility of MEAs in different settings was classified into three categories: (1) feasible, (2) not feasible, and (3) feasible but not obvious. Two authors independently performed the categorization within the feasibility analysis. Discrepancies were resolved through discussion until consensus was reached.

## 3. Results

### 3.1. Literature Inclusion

In the systematic literature search, a total of 11,295 unique papers were identified, of which 97 were included in the full-text analysis. Thirty-three of these were ultimately excluded, while the reference search of the remaining 64 papers identified 18 additional relevant articles. The final included cohort therefore consisted of 82 papers. The inclusion flowchart of the systematic literature search can be found in Appendix B. Of the 82 included studies, 61 related specifically to drugs, 5 solely to medical devices, and 16 did not specify their focus or related to both. Thirty-one studies had a perspective that was not specific for a country, while the rest focused on one or multiple countries. Only two of those included an explicit discussion of MEAs within a middle-income country, while none specifically discussed MEAs within a low-income country setting.

### 3.2. Clustered Managed Entry Agreements and Their Benefits and Limitations

The 82 papers described 117 MEAs. Many of those were identical or similar and thus clustered following the process described in the methods section. This resulted in a set of 15 unique MEAs, each including one or more similar MEAs. Of these 15, four entailed payment structures. An overview of the 15 MEAs, along with a short description of how they function can be found in Table 1. Literature described the MEA as being in use in at least one country for 12 of the 15 included MEAs, while for three MEAs, the literature described only a conceptual proposal, namely cost-plus pricing, two-part pricing, and patent buyout. The benefits and limitations of each clustered MEA as extracted from literature are listed in Table 2.

### 3.3. Feasibility Assessment

A graphical overview of the feasibility of the included MEAs in different settings can be found in Table 3. Most agreements can be applied throughout the different settings that we assessed and can be applied in combination with multiple other agreements and in different payment structures. However, some limitations apply. For instance, subscription payment structures inherently delink value and quantity and, therefore, cannot easily be combined with agreements that have a value- or outcome-based element. Similarly, upfront payment is feasible with a pay-per-outcome scheme, as it is possible to implement rebates when an outcome is not achieved, but it makes more sense to organize payment at outcomes achieved.

Literature described that almost all MEAs could be applied to one-time as well as to chronic treatments, though coverage with evidence development and pay-per-outcome agreements may be less obvious for one-time treatments because of the long time horizon associated with measuring outcomes and developing evidence. It is still feasible to implement these agreements—for example by replacing upfront payment with annuity payments linked to patients achieving certain outcomes or the manufacturer progressing with evidence generation—but linking multiple complicated MEAs may not be the most obvious choice, as managing them is very labor intensive and costly [1,62,66,69,81].

A central authority makes some MEAs easier to implement. With evidence generation usually being a global process, it is more obvious to organize such a scheme via a central authority (preferably in an international cooperative effort). Most countries aim to provide all patients with equitable access to therapies. A decentral budget threshold or dedicated fund can lead to some patients in a country having access while others do not, which is why it makes more sense to implement these two MEAs only when a central authority can manage them (e.g., the Cancer Drug Fund in the United Kingdom).

Most MEAs can be combined with one or multiple others, but some MEAs are mutually exclusive. For example, it is difficult to combine a patent buyout with most agreements because a private monopolist price does not apply anymore after a buyout. Some combinations are feasible but not obvious. The combination of cost-plus pricing with two-part pricing is such a combination, because the formation of a price for a therapy based on production costs leaves little room for an access fee.

## 4. Discussion

As demonstrated, payers have many options to enter into MEAs with manufacturers. Because most agreements can be applied in several different ways and can be combined with other agreements, the possibilities for constructing tailored reimbursement mechanisms are extensive. Through these mechanisms, payers (be it governments, insurers, health technology assessment organizations, healthcare providers, or others) can manage the financial risks associated with funding expensive, innovative therapies.

MEAs are most potent when they are tailored to the specific circumstances in which they are being used and to the preferences of the decision-maker in question. This tailoring should include a careful consideration of the characteristics of the therapy to be reimbursed, the characteristics of the healthcare payment system, the possibilities for different payment structures, and preferences of the decision-maker regarding financial vs. outcome-based agreements, individual or population-based agreements, and the combination of multiple mechanisms. Our comprehensive analysis of the feasibility of MEAs in each of these settings can help guide the case-by-case selection of a combination of agreements.

The identified benefits and limitations of the MEAs may seem contradictory. However, this observation relates to the various perspectives or interests of stakeholders involved [82,83,84,85,86]. Restrictive reimbursement for a subgroup, for example, always has multiple perspectives, i.e., loss of revenue for the company or lack of access for a specific patient population, but an opportunity for an HTA organization or payer to gain evidence that is lacking.

### 4.1. Clustering of Agreements

Some MEAs are similar but at the same time distinctly different. Therefore, in some studies, they are clustered, while in others they are treated separately [1,2,3,4,52,81]. For example, volume–price agreements and budget thresholds are sometimes treated as a similar agreement, because both consider the volume (either in units or in costs) and link it to the price. We have treated them separately because a distinction is that price–volume agreements are usually progressive and do not have a cutoff after which additional volume leads to a price of zero, while budget thresholds inherently stop funding after a certain threshold, irrespective of whether this means the company will provide the drug for free, patients will have to pay for it themselves, or something else. Alternatively, we clustered budget thresholds and dedicated funds, as they both imply a certain limited amount of funding, while others have treated them separately [82].

We also regarded value-based pricing and pay-per-outcome schemes as similar but not the same. In general, value-based pricing includes a consideration of all aspects of value deemed relevant. For example, in oncology, the relative benefits of the treatment in improving quality of life and lengthening progression-free survival and/or overall survival may be considered, while correcting for possible side effects. Often, long-term effects are extrapolated to time horizons beyond those of the available data. Though it is possible to combine multiple outcomes in a pay-per-outcome scheme, usually one specific outcome is selected on which the pricing and payment is based [83]. Thus, the scope of pay-per-outcome schemes is often different from that of value-based pricing.

### 4.2. Implementation

The included literature described that none of the reimbursement mechanisms were used as standalone tools. By combining a pricing plan with additional agreements (e.g., evidence development obligations) and fitting a payment structure to these agreements, a comprehensive reimbursement mechanism can be constructed [3,48]. Though literature was most extensive for drugs, we found no indication that any of the MEAs could be applied only to drugs and not to other technologies.

For the same therapy, differences between healthcare systems could result in different suggestions for reimbursement mechanisms. A striking example is provided by novel hepatitis C treatments, the first of which (sofosbuvir) was approved in 2013 by the U.S. Food and Drug Administration and the European Medicines Agency. A course of sofosbuvir was so expensive that researchers calculated it would economically be more attractive for the United States government to buy the company rather than pay for individual treatments [27]. Australia (and others, e.g., the state of Louisiana in the U.S.) has implemented a subscription scheme (sometimes called a Netflix-model) for hepatitis C treatments. It entails unrestricted use of these drugs for an annual tariff [87]. Inherent to health leasing or subscription models is that the annual tariff is not linked to the value the therapy provides, since use is unlimited. The tariff may nevertheless be based on an estimation of the use and the prospected benefits, and retrospectively it may be feasible to calculate whether the system as a whole has been cost effective. The suitability of such a scheme within a healthcare system depends on the legal opportunities and the ability of payers to enter into it. For systems with the ability to enter into such schemes, the willingness of payers and manufacturers to partake depends on whether the tariff provides a reduced and/or predictable financial burden for a greater treated population for payers and a sufficient and predictable return on investment for manufacturers [87]. The evaluation, reporting, and improvement of such schemes is crucial for future successes with them in different settings [1].

Pay-for-performance is often used as a synonym for outcome-based or performance-based agreements and it works as an umbrella term for any agreement that includes an outcome element [88]. Pay-for-performance is usually referred to as a combination of a pricing, usage, and payment model, i.e., it suggests there exists an agreement on the type of patients eligible for treatment, what is going to be paid for the outcomes that these patients may or may not achieve, and when this payment is due. In practice, this means that dozens of optional reimbursement mechanisms all fall within the scope of pay-for-performance [1]. In this study, we have explicitly split the separate pricing and payment elements within a pay-for-performance scheme to highlight these possible combinations. The implementation of performance-based MEAs has been the subject of a recent study by the OECD [1]. It found that many countries are experimenting with performance-based MEAs, but results have been mixed and inconsistently reported. Most countries do not systematically evaluate the performance-based agreements that they implement. For those that have been evaluated, one striking finding is that coverage with evidence generation and pay-for-outcome schemes has often not resulted in resolving the uncertainties they were meant to resolve. The findings in the OECD report underscore our findings insofar that the feasibility assessment of pay-per-outcome in combination with coverage with evidence generation agreements concluded that it is not an obvious mix because it is difficult to upfront define a price for an outcome that is still surrounded by a lot of uncertainty. The OECD report further mentions that the administrative burden of performance-based MEAs can make them costly to execute [1]. A major drawback of pay-for-outcome schemes is the added burden to both the patient and the healthcare system to monitor the performance of the therapy. These costs can become so high that they outweigh any savings made by such a scheme [3,5,28,49,54,59,64]. The enforcement of additional studies to resolve the uncertainties regarding long-term effects could sometimes also be achieved by implementing a form of coverage with evidence development where it is agreed that the price will drop iteratively by a certain percentage or amount after predefined periods when the manufacturer does not produce the necessary evidence [89]. Such a reimbursement mechanism mitigates financial risks and enforces additional evidence generation while simultaneously preventing overly complicated outcome-based schemes. Based on our and previous results, payers are advised to develop a decision process that defines when it is worthwhile to implement a reimbursement mechanism consisting of a combination of multiple, complicated MEAs and when it may be more appropriate to implement a simpler reimbursement mechanism.

Besides the MEAs between payers and manufacturers described in this study, payers may want to install additional mechanisms irrespective of their agreement with the manufacturer. For example, in the case of high one-time costs for a very rare disease in a multi-payer system, payers can agree amongst themselves to construct a high-risk pool from which those that have to reimburse the treatment can acquire funds [60,75]. Similarly, payers may want to regulate copayments by patients to stimulate expedient use and reduce budget impact [38].

### 4.3. Higher- vs. Lower-Income Settings

Our literature review yielded only a few papers specifically describing MEAs in a non-high-income setting. Though theoretically most MEAs could be applied in any setting, and it is very likely that countries with more limited resources could benefit more from applying them, previous research has shown that some MEAs are actually used less in lower-income settings [1,30]. Particularly the more complex MEAs that require more resources, such as pay-per-outcome schemes (that need monitoring of treatment effects) and value-based pricing agreements (that require an extensive health technology assessment process to be completed), are more difficult to implement in countries with fewer resources. The adoption of some MEAs may even be detrimental to the introduction of innovative therapies in lower-income settings. International (external) price referencing has been criticized for leading to higher drug prices in lower-income countries and for not delivering adequate solutions in cases where the referenced countries are themselves using MEAs (thereby concealing the actual price they pay for drugs) [12,90]. More research is needed to identify which MEAs are currently being applied, to what extent certain MEAs are more or less feasible in lower-income settings, and how barriers for their implementation could be overcome.

### 4.4. Limitations

We excluded reimbursement mechanisms that were not based on an agreement between a payer and the manufacturer. This corresponds to previous literature describing MEAs [1], but it also means we excluded some mechanisms that may be relevant for payers in funding innovative treatments (e.g., high-risk pools between payers, bundled payment agreements between hospitals and payers). Irrespective of the agreements payers make with manufacturers, payers should consider what other activities they can employ to mitigate financial risks and enhance value in healthcare. While a comparison with other reports on MEAs [1,2,3,4] suggests that all relevant agreements were identified, mechanisms for which no or very few publications exist may not be included in this review. Additionally, a search of the gray literature may have yielded additional insights regarding the feasibility of the different MEAs. As discussed, our categorization of MEAs is not absolute. Some MEAs are considered to be more or less similar depending on the perspective of the reader. It remains important for payers to study the given definitions, benefits, and limitations before accepting the analysis we provide in Table 3. The benefits and limitations of each MEA may differ to some extent based on the stakeholder group that reports them. A detailed analysis of each stakeholder’s opinion on all MEAs would be insightful as it can highlight situations with opposing interests, but it goes beyond the scope of this study.

## 5. Conclusions

For successful and sustainable reimbursement of expensive innovative therapies, payers may enter into a combination of multiple MEAs with the manufacturer. Many options for combining MEAs into a reimbursement mechanism exist. Such reimbursement mechanisms should be tailored to the type of treatment, healthcare payment setting, and preferences of the decision-maker regarding the simplicity of the scheme, application on an individual or population level, and the payment structure.

## Figures and Tables

**Table 1:** Overview and description of identified managed entry agreements.

Agreement	Description
Price–volume agreements	Drug prices are progressively lowered as more patients receive the treatment.
Budget threshold/dedicated funds	Maximum amount of spending for an individual innovative treatment (budget threshold) or therapeutic area (dedicated funds) to contain total expenditures. Translates into maximum number of patients treated per year or sharing of costs with the manufacturer or patients after costs have been exceeded.
Discounts/rebates	Simple price discounts, publicly or confidentially agreed upon between the payer and manufacturer.
Patent buyout/direct funding	Acquisition of the intellectual properties protecting a therapy globally or within a jurisdiction.
Cost-plus pricing	Fixed-price covering costs for producing and distribution of a therapy while allowing some profit to be made. Research and development costs can be integrated into this price and the profit margin can be linked to the value a therapy provides.
Two-part pricing	Dividing the price of a product into an entry fee and usage price. Paying the entry fee gives buyers the right to buy the product at the usage price, which is substantially lower than the price a monopolist would charge. The entry fee can be calculated based on the value a therapy provides.
Reference pricing	External reference pricing: the practice of using the price(s) of a medicine in one or several countries in order to derive a benchmark or reference price for the purposes of setting or negotiating the price of the product in a given country. Internal reference pricing: a reimbursement policy in which identical medicines (ATC 5 level) or similar medicines (ATC 4 level) are clustered (reference group).
Value-based pricing	Policy to set the prices of a new medicine and/or decide on reimbursement based on the (societal) therapeutic value that a therapy offers, usually assessed through health technology assessment (HTA). To compare value across healthcare domains incremental cost-effectiveness ratios and willingness-to-pay thresholds can be used.
Pay-for-outcome/outcome guarantees	The price level and/or revenue received is related to the future performance of the product in either a research or real-world environment. Therapy costs are eliminated or reduced by the manufacturer if outcomes are not achieved.
Conditional treatment continuation/risk sharing	Continuation of coverage for individual patients is conditioned upon meeting short-term treatment goals.
Coverage with evidence development	Provisional reimbursement of promising technologies with limited clinical evidence. Temporary reimbursement is granted with an obligation for the manufacturer to obtain and provide additional data. Can be organized either with patients only having access when included in the study (only in research) or with an obligation to generate data and unrestricted access (only with research)
**Payment structures**	**Description**
Upfront payment	Paying treatment costs upfront at the time of delivery of treatment. Can be combined with rebates when a therapy does not achieve predefined outcomes.
Pay at outcomes achieved	Paying treatment costs only after results have been achieved.
Annuity payments/over-time payments/staggered payment	Spreading payments over multiple years, with an agreement upon amount of treatment or outcomes delivered.
Health leasing/subscription	Paying for unlimited use of a therapy during a predefined period.

**Table 2:** Benefits and limitations of each of the included managed entry agreements. QALY: quality-adjusted life years, HTA: health technology assessment, ICER: incremental cost-effectiveness ratio.

Agreement	Benefits	Limitations
Price–volume agreements	Places a limit on maximum expenditure per drug while ensuring availability to patients [14,15] Effective cost-control mechanism when limited measures are available to prevent off-label prescribing or prescribing in populations where the drug will be less cost effective [5,16]	Unsure how likely manufacturers are to adopt this system [14] Financially unpredictable for companies. More sales may lead to lower revenues [15] Manufacturers may purposefully estimate expected users wrongfully to maximize profit [17] Does not always consider issues such as compliance [5]
Budget threshold/dedicated funds	Straightforward way of limiting total drug expenditure while allowing access to innovative medicines [14,15,18,19] Knowledge of potential maximum returns may incentivize manufacturers to be more tactical in choosing their investments, improving efficiency of the industry [3] Dedicated funds can provide patients with access to therapies they would not have had otherwise [20]	May be complex to apply in real-word regulatory settings [5,14] Could lead to high copayments or loss of access for patients when they need the drugs after the threshold is met [15,18,19] Can work against the incentives created by value-based pricing, by limiting attainable profits for manufacturers to earn back investments [3] In a volume-based threshold system, manufacturers may raise the price to gain as much profit as possible under the threshold [21] Dedicated funds are at risk of overspending or, if overspending is prohibited, will default in providing access to patients [20]
Discounts/rebates	Simple and proven effective way to reduce prices and budget impact [22,23]	Confidential discounts make it difficult to assess whether therapies deliver value for money [22,24] Creates imbalance between payers and manufacturers as payers do not know what other payers are paying while the manufacturer has all the information [24,25] Big and developed countries may have an advantage in negotiating better prices, increasing the imbalance in access to therapies between higher- and lower-income countries [23,25,26]
Patent buyout/direct funding	Can save total costs for payers in cases where target populations are large [27] May act to incentivize innovation by guaranteeing profits for manufacturers/developers and government can also gain part of the profits made [28] Governments have lower cost of capital than companies, so they can loan the needed money at a more favorable rate [3] Could be organized through tax credits [29]	The entity responsible for the patent buyout must be willing to make a large one-time investment without a clear profit prospect [27,28] Manufacturing must still be organized and paid for [28] The transfer of a patent can have global effects on accessibility [30]
Cost-plus pricing	Easy to implement [31,32,33] Compensating manufacturers for costs made in development incentivizes innovation [3]	Effect on price is highly dependent on cost definition. Payers have no direct knowledge of manufacturer costs [3] May not reward high research and development costs made by manufacturers, so does not incentivize innovation [31,32,33,34] Low-value drugs with high development costs may yield high price with low benefit to society [3]
Two-part pricing	Higher-volume payers will end up with lower average cost per usage than lower-volume payers [35]	Difficult to control for reselling of products [35]
Reference pricing	Creates price-limiting forces in monopoly-based markets, such as pharmaceuticals [12,13,34] A combination of internal and external reference pricing methods can be applied [34,36,37]	Reference pricing is unfeasible in systems with many independent private payers [38] Confidential rebates cause inaccurate reference prices [13] Easier to apply with generics than with newly developed drugs [36] When the countries that are used to reference the prices have different economies, the reference price may not be accurate [13,37] Reference pricing can make drugs unafforable for lower-income countries [12,13,37,39] Reference pricing is a blunt instrument that delinks additional costs and additional value [40]
Value-based pricing	Encourages innovations that provide actual benefit to society [3,33,41,42,43,44] Price is linked to the willingness-to-pay threshold [12,31,33,41,45,46] Cost offsets in sectors other than healthcare can be included in value calculation [47] Provides incentives for manufacturers to develop technologies that are more likely to be of value [41,44,45,48] Targets scarce resources to most cost-effective technologies [3,13,49]	Costs of pharmaceuticals may increase due to manufacturers setting their prices near willingness-to-pay thresholds [31,32,33,41,42,45] Difficult to introduce orphan drugs with the same standards as regular medicines [32,33], but simultaneously these standards are argued as sufficient given existing statutory benefits under orphan drug legislation [50] Value is often predicted at earlier stages of development, so may not accurately predict real-word value [51,52] Drugs are often used for multiple indications, all with different values. Complex to establish one price. The same is true in a system with multiple payers who have different preferences [33,41,53] Very effective drugs with high value can increase total costs when the life expectancy of patients increases [33,41,48] Risk of non-return on investment is transferred completely to payer at time of payment [52] High upfront costs to payers needing to calculate or define value-based prices [38] Value predictions are not perfect and may not capture all elements of value [3,49,54,55] Difficult to establish the value of diagnostic procedures [43,51] QALY-based pricing may discriminate against patients with a short life expectancy [56] Difficult to compare cost-effectiveness studies across different countries [57]
Pay-for-outcome/outcome guarantees	Lowers drug costs compared to full payment [58,59] Allows patients access to innovative medicines despite uncertainty of clinical benefit and cost effectiveness [15,16,58,60,61] Incentive for manufacturers to develop better diagnostics to improve effectiveness in treated populations [5,62] Improves alignment of reward to the manufacturer with value patients would assign to the treatment [1,3] Reduces the likelihood of payers indefinitely using technologies that are not cost effective [63]	High administrative burden leading to extra costs may offset cost gains [1,3,5,28,49,54,59,63,64,65] Can be difficult to measure outcomes in curative therapies, due to the time lag between administration and apparent clinical benefit. In general, it is hard to establish and reliably measure effectiveness due to changes over time in clinical practice [1,3,5,28,49,54,59,64] Pricing regulations can interfere with outcome-based payment schemes [60,61] Is difficult to implement in a system with competition between multiple insurers [38,66] Could be less appropriate with very small patient populations [62] Applicability is limited to a small subset of therapies [65,67]
Conditional treatment initiation/continuation	Facilitates appropriate use of therapies [1,2,3,68] Can mitigate the risks associated with uncertain effects in certain patient populations [1,2,3]	Can require constant monitoring, making this approach resource intensive [1,2,3] The stakeholder benefiting financially may be different from the stakeholder who makes the extra effort to monitor, making successful implementation more difficult [1,3,69]
Coverage with evidence development (CED)	Allows patient access to new medical products during development. Early involvement of HTA bodies reduces uncertainty [49,56,70,71] Effective method of handling cost uncertainty and availability when only patients involved in research are allowed access [52,71] Initial high ICER was reduced substantially after additional research [71] By linking payment to evidence development, manufacturers have an incentive to conduct more research [34] Can also be used for innovative diagnostic procedures [72]	Patient access may be terminated after the CED scheme [70,71,73] National CED research can be redundant considering international research by manufacturers [70,73,74] Restricts access to effective treatments [52] Reduces return on investment in developing new technologies [52] Performing extra studies to prove cost effectiveness leads to more costs [16,52,56,71] Public pressure can force payers to allow more patients access to the treatment than included in evidence development schemes [34] Payers are more keen to pay for established therapies, instead of for additional research [34] Difficult to align the standards for study design and resulting lack of clarity whether data generated under CED schemes is sufficient for making coverage decisions [1,74]
**Payment mechanism**	**Benefits**	**Limitations**
Upfront payment	Easiest mechanism to implement [7,28] Rebates may be able to mitigate some of the risks associated with upfront payment [7,28]	High financial burden on payers with high associated risk [7,28]
Payment after achieved outcomes	Relatively little risk for payers [1,2,28,58,59]	High financial burden on manufacturers with high associated risk [1,2,28,58,59]
Annuity payments	Spreading out high costs over longer period of time assures that more patients per year can be treated within the same yearly budgets [2,3,7,28] Acceptable mechanism for industry and payers [60,75,76] Possibility to create a liquid healthcare-loan market with substantial leverage for payers and lenders to negotiate prices [77] Can be combined with risk-shared contracts to set the duration of payment [76,78]	Does not lower the price of treatment unless combined with other measures, and interest rates may apply, which would make the total price higher [60] Requirement of diagnostic monitoring may add extra burden to providers [76,78] Difficult to implement in healthcare systems with multiple payers between which patients can switch [76,78] Unclear where initial financing will come from, especially with smaller insurers [61]
Health leasing/subscription	Reduces uncertainty for payers and manufacturers [79,80] Could prevent high upfront costs [79] Can facilitate the treatment of large patient populations for drugs with low production but high unit costs [79] Could reduce manufacturers’ incentives to aggressively market drugs [80]	Does not affect total budget impact of treatment per patient in all cases [79] Accurate forecasting may be difficult for payers as well as manufacturers, making it hard to establish an appropriate financing limit [79] For manufacturers, time to return on investment can be increased [80]

**Table 3:** Applicability and feasibility of included managed entry agreements in different settings including an assessment of the feasibility of combinations of agreements.

	Price–Volume Agreements	Budget Threshold/Dedicated Funds	Discounts/Rebates	Patent Buyout	Cost-Plus Pricing	Two-Part Pricing	Reference Pricing	Value-Based Pricing	Pay-for Outcome/Outcome Guarantees	Conditional Treatment Continuation	Coverage with Evidence Development
Categorization											
Outcome-based											
Purely financial											
Individual patient level											
Target population level											
Combination with payment scheme											
Upfront payment											
Payment at outcome achieved											
Annuity payment											
Health leasing/subscription											
Type of treatment											
One-time treatments											
Chronic treatments											
Healthcare payment system											
Central authority											
Decentral authority											
Combination matrix											
Price–volume agreements											
Budget threshold/dedicated funds											
Discounts/rebates											
Patent buyout											
Cost-plus pricing											
Two-part pricing											
Reference pricing											
Value-based pricing											
Pay-for outcome/outcome guarantees											
Conditional treatment continuation											
Coverage with evidence development											
	Feasible										
	Feasible but not obvious										
	Not feasible										

**Figure A1:**
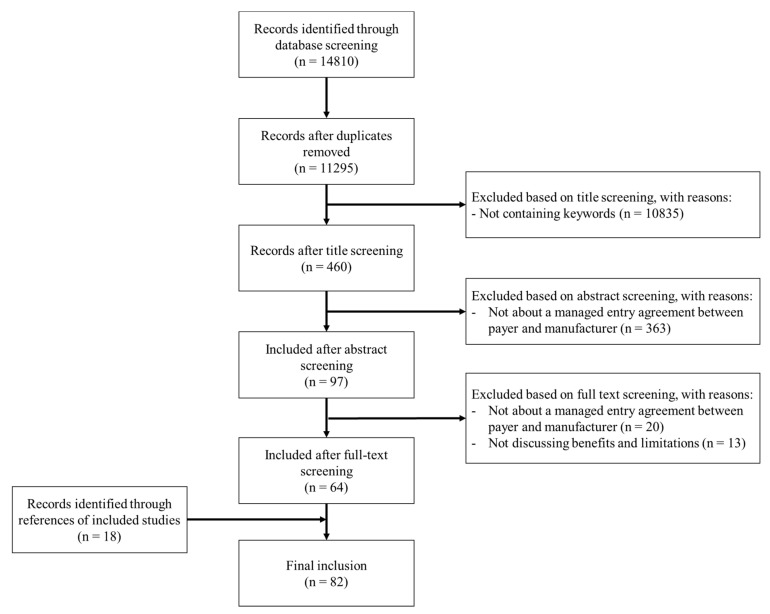
Flowchart for the inclusion of studies that described managed entry agreements.

## Appendix A. Search Strategy

### Appendix A.1. PubMed Search String

((Economics, pharmaceutical [mh] OR economics, medical [mh] OR insurance, health, reimbursement [mh] OR costs and cost analysis [mh]) AND (innovat* [tiab] OR new [tiab] OR Cell- and Tissue-based therapy [mh] OR Genetic Therapy [mh]) AND (reimburs* [tiab] OR pric* [tiab] OR payment [tiab] OR insurance [tiab] OR afford* [tiab])) AND (“2000”[Date—Publication]: “3000”[Date—Publication]).

### Appendix A.2. Embase Search String

(‘pharmacoeconomics’/exp OR ‘reimbursement’/exp OR ‘health care cost’/exp) AND (‘innovat*’:ab,ti,kw OR ‘new’:ab,ti,kw OR ‘biological therapy’/exp OR ‘biological therapy’) AND (‘reimburs*’:ab,ti,kw OR ‘pric*’:ab,ti,kw OR ‘payment’:ab,ti,kw OR ‘insurance’:ab,ti,kw OR ‘afford’:ab,ti,kw) NOT ‘conference abstract’/it AND [2000–2019]/py.

### Appendix A.3. Keywords Used in Title/Abstract Screening

Managed entry agreement, reimbursement, payment, pricing, policy, cost, price, innovative, atmp, new, gene/cell therapy, expensive, payer, financing.
